# The effect of nitrate and phosphate availability on *Emiliania huxleyi* (NZEH) physiology under different CO_2_ scenarios

**DOI:** 10.3389/fmicb.2013.00155

**Published:** 2013-06-18

**Authors:** Mónica Rouco, Oscar Branson, Mario Lebrato, M. Débora Iglesias-Rodríguez

**Affiliations:** ^1^National Oceanography Centre Southampton, School of Ocean and Earth Science, University of SouthamptonSouthampton, UK; ^2^Department of Biology, Woods Hole Oceanographic InstitutionWoods Hole, MA, USA; ^3^Department of Earth Sciences, University of CambridgeCambridge, UK; ^4^GEOMAR. Helmholtz Centre for Ocean Research KielKiel, Germany; ^5^Department of Ecology, Evolution and Marine Biology, University of California Santa BarbaraSanta Barbara, CA, USA

**Keywords:** *Emiliania huxleyi*, ocean acidification, nutrients, alkaline phosphatase, nitrate reductase, calcification

## Abstract

Growth and calcification of the marine coccolithophorid *Emiliania huxleyi* is affected by ocean acidification and macronutrients limitation and its response varies between strains. Here we investigated the physiological performance of a highly calcified *E. huxleyi* strain, NZEH, in a multiparametric experiment. Cells were exposed to different CO_2_ levels (ranging from 250 to 1314 μatm) under three nutrient conditions [nutrient replete (R), nitrate limited (-N), and phosphate limited (-P)]. We focused on calcite and organic carbon quotas and on nitrate and phosphate utilization by analyzing the activity of nitrate reductase (NRase) and alkaline phosphatase (APase), respectively. Particulate inorganic (PIC) and organic (POC) carbon quotas increased with increasing CO_2_ under R conditions but a different pattern was observed under nutrient limitation. The PIC:POC ratio decreased with increasing CO_2_ in nutrient limited cultures. Coccolith length increased with CO_2_ under all nutrient conditions but the coccosphere volume varied depending on the nutrient treatment. Maximum APase activity was found at 561 μatm of CO_2_ (pH 7.92) in -P cultures and in R conditions, NRase activity increased linearly with CO_2_. These results suggest that *E. huxleyi*'s competitive ability for nutrient uptake might be altered in future high-CO_2_ oceans. The combined dataset will be useful in model parameterizations of the carbon cycle and ocean acidification.

## Introduction

Since the beginning of the industrial revolution, atmospheric CO_2_ has increased at the fastest rate experienced by the Earth in the last 65 million years (Zachos et al., 2001). Consequently, the increase in CO_2_ dissolution in seawater has been altering the balance of the inorganic carbon species leading to a decrease in pH predicted to intensify over the next century (Raven et al., 2005). Additionally, global warming derived from an increase in greenhouse gases induces stratification of the water column reducing mixing processes that maintain nutrient supply into the euphotic zone (Sarmiento et al., 1998). Therefore, the duration and timing of nitrate and phosphate limitation and the biogeographical regions affected are likely to vary in future oceans (Sarmiento et al., 1998). Coccolithophores play a major role in the carbon cycling being an important source of calcite in the open ocean (Gehlen et al., 2007). *Emiliania huxleyi* is the most abundant bloom-forming coccolithophore species (Tyrrell and Merico, 2004), and its calcification process is known to be affected by variations in carbon chemistry (Riebesell et al., 2000; Iglesias-Rodriguez et al., 2008; Langer et al., 2009). However, this response varies if other environmental parameters such as nutrient availability, temperature or light are simultaneously changed (Zondervan et al., 2002; Sciandra et al., 2003; Feng et al., 2008; De Bodt et al., 2010; Borchard et al., 2011).

*E. huxleyi* flourishes after the demise of diatoms, when silicate, nitrate, and phosphate are limiting (Litchman et al., 2006). This ecological strategy stems from a low nutrient quota and an extremely high phosphate affinity under phosphate-limiting conditions (Riegman et al., 2000). *E. huxleyi* also takes up nitrogen compounds other than nitrate (Benner and Passow, 2010; Bruhn et al., 2010), and assimilates nutrients from organic sources through the controlled expression of enzymes active in specific metabolic pathways (Dyhrman and Palenik, 2003; Bruhn et al., 2010). Despite the sensitivity of *E. huxleyi* to CO_2_ (Riebesell et al., 2000; Iglesias-Rodriguez et al., 2008; Langer et al., 2009) and its diversity of nutrient acquisition pathways, the majority of contemporary work has not considered the effect of [CO_2_] on the efficiency of nutrient assimilation. Previous studies have independently assessed the biogeochemical responses of *E. huxleyi* to high CO_2_ under nitrogen (Sciandra et al., 2003; Leonardos and Geider, 2005; Müller et al., 2012) or phosphorus (Borchard et al., 2011) limitation using three different *E. huxleyi* strains (TW1 PML, B92/11 and a strain isolated in the Raunefjord, Norway). It is well accepted that different *E. huxleyi* strains respond differently to varying CO_2_ levels (e.g., Langer et al., 2009). For example, the *E. huxleyi* NZEH strain presents contrasting calcification responses to elevated CO_2_ compared to other strains tested in the laboratory. Similarly, a recent field study revealed the presence of a heavy calcified *E. huxleyi* morphotype (R-morphotype) in “acidic” waters of the South Pacific Ocean, as an exception to the global correlation found between coccolithophore calcification and CO^−2^_3_ concentration (Beaufort et al., 2011). Considering this biological variability and the projected changes in the extent of oligotrophic waters, studying the effect of nutrient limitation in conjunction with ocean acidification in different *E. huxleyi* strains is crucial.

In this study, we assessed the combined effects of elevated atmospheric CO_2_ and nitrate or phosphate limitation on the physiology of the *E. huxleyi* strain NZEH. This is a highly calcified strain bearing coccoliths that display the R-morphotype whose production appears to be resilient to ocean acidification (Iglesias-Rodriguez et al., 2008; Beaufort et al., 2011). We also investigated the effect of CO_2_ on nitrate and phosphate utilization by analyzing the activity of two enzymes involved in nutrient assimilation: alkaline phosphatase (APase), and nitrate reductase (NRase). Investigating the response of different *E. huxleyi* strains, and potential discrepancies between them, to environmental change is central to model the contribution of this ecologically important species to the global carbon cycle.

## Materials and methods

### Culture conditions

Experiments were conducted in diluted batch cultures of *E. huxleyi* (Lohmann) W. W. Hay and H. P. Mohler, strain NZEH (CAWPO 6), isolated in 1992 in the South Pacific Ocean and obtained from the Plymouth Culture Collection (UK). Artificial sea-water (ASW) was prepared according to Kester et al. (1967) with different nitrogen and phosphorus concentrations to achieve nutrient (nitrate, phosphate)-replete (R), nitrate-limited (-N) and phosphate-limited (-P) conditions (Table 1). Trials were conducted prior to the experiments to ensure that -N and -P cultures reached nutrient limitation at the desired cell density, a density below levels that would alter significantly the media carbon chemistry. The three nutrient regimes were combined with different CO_2_ partial pressures (ranging from 250 to 1314 μatm) corresponding to pre-industrial levels and projected values for the middle and the end of the century respectively (Table 1). Medium carbonate chemistry was adjusted by additions of sodium carbonate (Na_2_CO_3_) and hydrochloric acid (HCl) to change the relative proportion of dissolved inorganic carbon (DIC) species and restore total alkalinity (TA) respectively (Riebesell et al., 2010). The conditions mimicked changes in carbonate chemistry associated with ocean acidification (CO_2_ increases while TA remains constant at ~2268 ± 64.86 μmol kg^−1^) (Table 1). The culture medium was filtered through sterile 0.22 μm polycarbonate filters (Millipore® Stericup™ Filter Units). All other environmental parameters remained constant throughout the experiments: salinity = 34.00 ± 0.40, temperature = 19.00 ± 0.50°C, 12:12 h light:dark cycle, irradiance = 120.00 ± 15.00 μmol photons m^2^ s^−1^ under Sylvania Standard F36W/135-T8 white fluorescent lighting (Havells Sylvania, Newhaven, UK).

**Table 1:** **Nutrient concentrations and carbon chemistry parameters of the media at the beginning and at the end of the experiment**.

	**Nutrient condition**	**[NO_3−_] (μmol kg^−1^)**	**[PO_43−_] (μmol kg^−1^)**	**DIC (μmol kg^−^)**	**TA (μmol kg^−^)**	**pH_total_**	**pCO_2_ (μ atm)**	**[HCO_3−_] (μmol kg^−^)**	**[CO_3−_] (μmol kg^−^)**	**CO_2_ (μmol kg^−^)**	**Ω-Ca**
Initial^a^	R	161.2	3.5	1906	2230	8.18	**258**	1674	222	8	5.3
End^b^		149.0 (10.4)	3.0 (0.1)	1808 (5)	2141 (4)	8.21 (0.00)	225 (2)	1575 (5)	225 (1)	8 (0)	5.4 (0.0)
Initial	R	154.4	4.1	2040	2221	7.90	**555**	1890	132	19	3.2
End		147.6 (0.9)	2.6 (0.0)	1937 (65)	2113 (7)	7.91 (0.01)	519 (9)	1793 (5)	127 (2)	17 (0)	3.0 (0.0)
Initial	R	156.2	3.4	2238	2330	7.67	**1073**	2117	86	36	2.1
End		149.4 (0.9)	1.7 (0.1)	2102 (14)	2179 (12)	7.64 (0.01)	1080 (27)	1990 (14)	75 (1)	36 (1)	1.8 (0.0)
Initial	-N	3.7	3.6	1892	2221	8.19	**250**	1658	225	8	5.4
End		0.3 (0.1)	2.9 (0.4)	1734 (4)	2021 (3)	8.16 (0.01)	251 (5)	1534 (5)	192 (3)	8 (0)	4.6 (0.1)
Initial	-N	3.1	3.5	2085	2308	7.99	**464**	1909	161	16	3.9
End		0.1 (0.0)	2.9 (0.0)	1917 (4)	2111 (5)	7.95 (0.00)	465 (4)	1765 (3)	137 (1)	16 (0)	3.3 (0.0)
Initial	-N	3.3	3.5	2195	2264	7.60	**1229**	2082	72	41	1.7
End		0.1 (0.0)	1.6 (0.00)	1958 (30)	1994 (1)	7.51 (0.01)	1358 (25)	1860 (3)	52 (1)	45 (1)	1.3 (0.0)
Initial	-P	158.8	0.2	1897	2217	8.18	**256**	1667	222	9	5.3
End		156.7 (0.21)	0.0 (0.0)	1790 (7)	2097 (8)	8.18 (0.00)	243 (3)	1573 (7)	209 (2)	8 (0)	5.0 (0.0)
Initial	-P	159.5	0.2	2169	2363	7.92	**561**	2004	147	19	3.5
End		153.3 (1.5)	0.0 (0.0)	1890 (3)	2109 (8)	8.01 (0.01)	394 (14)	1723 (5)	154 (5)	13 (1)	3.7 (0.1)
Initial	-P	152.7	0.2	2186	2241	7.57	**1314**	2075	67	44	1.6
End		151.5 (0.2)	0.0 (0.0)	2098 (3)	2153 (3.3)	0.57 (0.00)	1253 (8)	1992 (3)	64.8 (0.4)	42 (0)	1.6 (0.0)

a Average blank values at the beginning of the experiment, before the inoculation of the cells.

b Average values from the triplicates at the end of the experiment/values in brackets correspond to the standard deviation from the triplicates at the end of the experiment

***R***, nutrient replete; ***-N***, nitrate limited; ***-P***, phosphate limited conditions.

### Incubation experiments

Experiments were conducted in triplicate in 4 L Nalgene® polycarbonate bottles. After the cell inoculation at an initial density of 100 cells ml^−1^, the bottles were completely filled to minimize headspace, closed and sealed with Parafilm® until harvested. A blank control bottle (containing no cells) was incubated alongside each treatment. A fourth replicate bottle (seeded with the same original stock culture and at the same concentration as that used in the triplicate bottle experiments) was used for daily monitoring of cell density, temperature, pH and irradiance, to avoid opening any of the triplicate bottles during the course of the experiment. Cell densities at the time of harvest in R cultures were 75,988 ± 13,159 cells ml^−1^ depending on the treatments. The R cultures were harvested during exponential growth phase and did not experience nutrient limitation over the course of the experiment. The -N and -P cultures were harvested two days after exponential growth stopped (assessed by daily cell counts from the test bottle). This allowed cultures to be in growth-limiting conditions for 2 days (cell densities at the time of harvest were 71,587 ± 9250 and 43,288 ± 14,651 cells ml^−1^ for -N and -P cultures respectively). All cultures were allowed to grow for 8–10 generations, corresponding to a maximal DIC consumption of 12%. This number of generations ensured that almost 100% of the cells in the cultures experience the study conditions. At time 0 (pre-inoculation) and during harvesting (always conducted 3 h after the beginning of the light phase), samples were collected from all experimental bottles for analysis of carbon chemistry and macronutrient concentration in the medium, particulate organic carbon (POC), Ca^2+^ measurements [for determination of particulate inorganic carbon (PIC)], particulate organic nitrogen (PON) and phosphorous (POP), cell density, and scanning electron microscope (SEM) imaging. Samples were also collected for APase and NRase assays.

### Determination of nitrate reductase activity

Aliquots of 400 ml were centrifuged (2000 g, 4°C, 15 min), and the resulting pellets were snap frozen in liquid nitrogen and stored at −80°C. NRase was extracted by adding 500 μ l of a solution containing 0.20 M phosphate buffer (*pH* = 8.20), 1 mM dithiothreitol (DTT) and 0.50 M methylenediaminetetra-acetic acid (EDTA) to each pellet. The resuspended material was sonicated on ice for nine 10-s bursts (30 s intervals between bursts) using a VC300 Vibracell sonicator (Sonics and Materials, USA) with a 20-kHz frequency, 50% duty cycle and an output of 3 (90 W). The final extract was centrifuged again (750 g, 4°C, 5 min), and the supernatant was used for the enzyme activity determination. NRase assays were developed according to Rigobello-Masini et al. (2006). Tests were carried out in triplicate in 1 ml at 19°C. The reaction mixture contained 100 μ l of crude extract, 10 mM KNO_3_ and 2 mM MgSO_4_ and was initiated by the addition of reduced nicotinamide adenine dinucleotide (NADH) substrate to a final concentration of 0.40 mM. The NRase reaction was stopped after 15 min with 250 μl of absolute ethanol at 0°C and with 50 mM ZnSO_4_. Activity was estimated based on the final nitrate concentration, indicated by the formation of a red AZO product after the simultaneous addition of 100 μ l (0.10% weight in volume, w/v) sulphanilamide and 100 μ l (0.10% w/v) *N*-1-naphtyl ethylenediamine dihydrochloride (Nicholas and Nason, 1957). After these additions, the reaction mixture was centrifuged again (21,000 g, 5 min), and the supernatant taken for colorimetric analysis at 543 nm. Absorbance values were converted to nitrate concentration using a calibration curve. Enzymatic activities were expressed in enzymatic units per total protein, where one unit of the enzyme activity (UEA) catalyzes the conversion of 1 μmol of nitrate to nitrite. Total protein determinations were performed with a commercial kit (Thermo Scientific Pierce® BCA Protein Assay Kit).

### Determination of alkaline phosphatase activity

APase is expressed on the cell surface of *E. huxleyi* (Dyhrman et al., 2006), allowing the activity assay to be performed on whole cells. Aliquots (40 ml) of sample were centrifuged at 2000 g and 19°C for 15 min, and the pellets were resuspended in 2.90 ml of a solution containing 0.01 M Tris buffer (*pH* = 9.00), 0.05 M MgCl_2_ and 0.01 M CaCl_2_. After resuspension, 100 μl of 13.50 M *p*-nitrophenilphosphate (*p*-NPP; Sigma) substrate were added, and the mixture was incubated at 19°C for 20 min. The reaction was stopped by the addition of 0.60 ml of 1 M NaOH, and samples were centrifuged again (3000 g, 19°C, 15 min) before the absorbance of the supernatant at 410 nm was measured. Absorbance values were transformed to *p*-nitrophenol (*p*-NP) concentration using a suitable calibration curve. One unit of enzymatic activity corresponds to 1 nmol of *p*-NP produced per 10^6^ cells min^−1^.

### Growth rate and coccosphere volume

Growth rate was determined with a standard exponential growth equation (Reynolds, 1984):

(1) $$\mu =(Ln\left(N_{t}\right)-Ln\left(N_{0}\right))/t$$

where *N*_0_ and *N*_*t*_ are the cell densities at the start and at the harvest day respectively, and *t* corresponds to the length of incubation (in days). Cell density and estimated coccosphere (cell + coccoliths) volume were determined in triplicates using a Beckman Coulter Multisizer III with a 70 μm aperture.

### Coccolith length

Coccolith length was measured from SEM images. For SEM sample collection, a 25 mm MF 300 filter was soaked with a drop of dilute ammonium hydroxide, and a 0.22 μm polycarbonate filter was placed on top. A few drops of culture were placed on the polycarbonate filter, and samples were dried on an open Petri dish (37°C for 24 h). A section of the top filter was cut out and sputter-coated in a Hummer VI-A gold coater, and a grid of 100 images at 5000× magnification was taken at a random location on each filter using a LEO 1450 VP SEM with SmartSEM V05-1 software. At least 60 coccoliths were measured on consecutive images along their longest axis (defined as coccolith length).

### Particulate matter analyses

POC and PON concentrations were measured using a Thermo Finnigan Flash EA1112 elemental analyzer with acetanilide standards at Plymouth Marine Laboratory (PML). Aliquots of 200 ml were filtered through two pre-combusted (400°C, 4 h) MF 300 filters (25 mm glass microfiber 0.70 μm pore size, Fisherbrand). Filters were kept at −20°C until required for analysis and fumed with sulphurous acid for 24 h in a desiccator chamber to remove inorganic carbon (Verardo et al., 1990). The filters were then dried at 60°C for 16 h and pelleted in pre-combusted aluminium foil (EMA; 100 × 30 mm circles) following Hilton et al. (1986).

For PIC analysis, 200 ml of medium was filtered through 0.20 μm 47 mm diameter Nuclepore polycarbonate filters, previously rinsed twice with 5 ml of dilute ammonium hydroxide solution (pH ~9), and washed again three times after filtering. Filters were stored in 50 ml Falcon tubes at −20°C until analysis. Samples (including blanks) were then weighed, and 15 ml of 0.10 M nitric acid were added and re-weighed to determine the acid volume. The filters were left in acid for 2–3 h with continuous shaking, after which 500 μl of the acid leach was removed and centrifuged (6500 g for 6 min). A 250 μl aliquot of the supernatant was taken to determine elemental concentrations in a Varian Vista Pro ICP-OES. The Ca^2+^ per coccolithophore was calculated and extrapolated to PIC, assuming that all Ca^2+^ on the filters originated in CaCO_3_ (Fagerbakke et al., 1994). The precision of the method, assessed from periodical measurements (*n* = 11) of standards was 1.17% RSD.

POP was measured using a wet-oxidation method, as described by Raimbault et al. (1999). Medium aliquots (200 ml) were filtered through a single pre-combusted (400°C, 4 h) MF 300 filter (25 mm glass microfiber 0.70 μm pore size, Fisherbrand). Samples were digested with sodium tetraborate and potassium persulphate and autoclaved before analysis in a Segmented Flow Auto Analyser (SEAL QuAAtro) at the National Oceanography Centre Southampton (NOCS), UK.

### Media chemistry

Samples (20 ml) of media were collected for nutrient measurements by filtration through a 0.22 μm Millex filter (Millipore, Billerica, MA, USA) and stored at −20°C until analysis. Macronutrient concentrations were determined colorimetrically following Hansen and Koroleff (1999) using a Segmented Flow Auto Analyser (QuAAtro, SEAL Analytical) at the NOCS (UK).

Samples for carbonate chemistry were collected in 300 ml borosilicate bottles and preserved in the dark with HCl at a final concentration of 2.5 10^−3^ M to prevent microbial growth during storage. These samples were later analysed to determine TA and DIC using a Verstatile INstrument for the Determination of Total inorganic carbon and titration Alkalinity (VINDTA3C) at the NOCS. DIC was analysed using a colorimetric titration (coulometer 5011, UIC, USA), and TA was determined using a semi-closed cell titration (Dickson et al., 2007). The precision of the method, assessed daily from repeated measurements (*n* ≥ 5) on the same batch of seawater, was 2.7 ± 1.6 μmol Kg^−1^ for DIC and 0.78 ± 0.78 μmol Kg^−1^ for TA. The accuracy was controlled against Certified Reference Materials (from A. G. Dickslon, Scripps Institution of Oceanography, USA) measured at the beginning and end of each day of analysis applying a correction factor obtained from the difference between the certified and the measured values. The carbonate system was calculated from temperature, salinity, DIC, TA and nutrients using the “CO2SYS” macro (Lewis and Wallace, 1998). The equilibrium constants were from Mehrbach et al. (1973) and refitted by Dickson and Millero (1987). The KSO_4_ constants were from Dickson (1990), and a seawater pH scale was used.

### Statistical analysis

One way factor ANOVA was conducted using SPSS 17 (SPSS Inc., Chicago IL, USA). Linear correlation factors (*r*^2^ value) were calculated using Sigma Plot 11.0 version (Systat Software Inc.).

### Limitation of the experimental approach

Our batch culture experimental design precludes a direct quantitative comparison of PON, POP, PIC, and POC quotas (pmol per cell^−1^) between R and -P or -N cultures. Unlike in R cultures, growth rates in the -N and -P treatments were not constant over the course of the experiment (see Langer et al., 2012; Langer et al., who used the same experimental approach). In nutrient-limited batch cultures, cells experienced an initial exponential nutrient-replete phase followed by a nutrient-limited phase when cell division rate decreased. Therefore, cellular quotas include cellular PIC and POC produced in both exponential and nutrient-limited periods. For this reason, these cannot be compared with cellular quotas under nutrient-replete conditions, where growth is constant and exponential during the experiment. Therefore, in this study, R, -P, and -N cultures are treated as separate experiments and comparisons of cellular PIC and POC quotas can be drawn between CO_2_ treatments but only within the same nutrient condition. Within each nutrient-limiting condition, the initial nutrient concentrations for all the CO_2_ conditions were identical (see Table 1). Given that in -N and -P experiments cells were harvested 2 days after the end of the exponential growth phase, any differences in PIC and POC quotas between the CO_2_ treatments (within each nutrient condition) are only the result of different CO_2_ levels.

## Results

### Enzymatic activity

**Figure 1:**
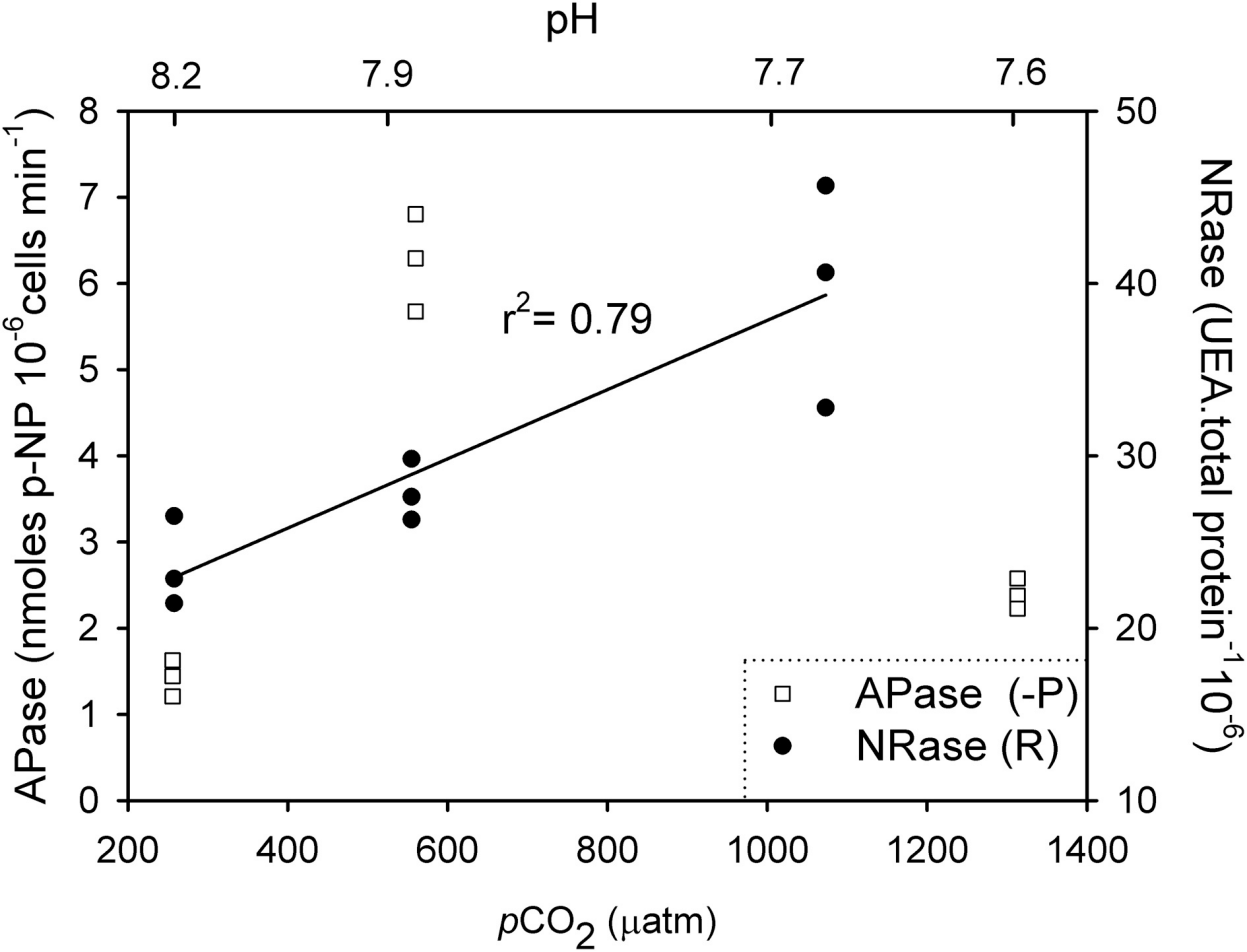
**Enzymatic response of nitrate reductase (NRase) and alkaline phosphatase (APase) to different CO_2_ and nutrient scenarios in *Emiliania huxleyi* NZEH**.

**Figure 2:**
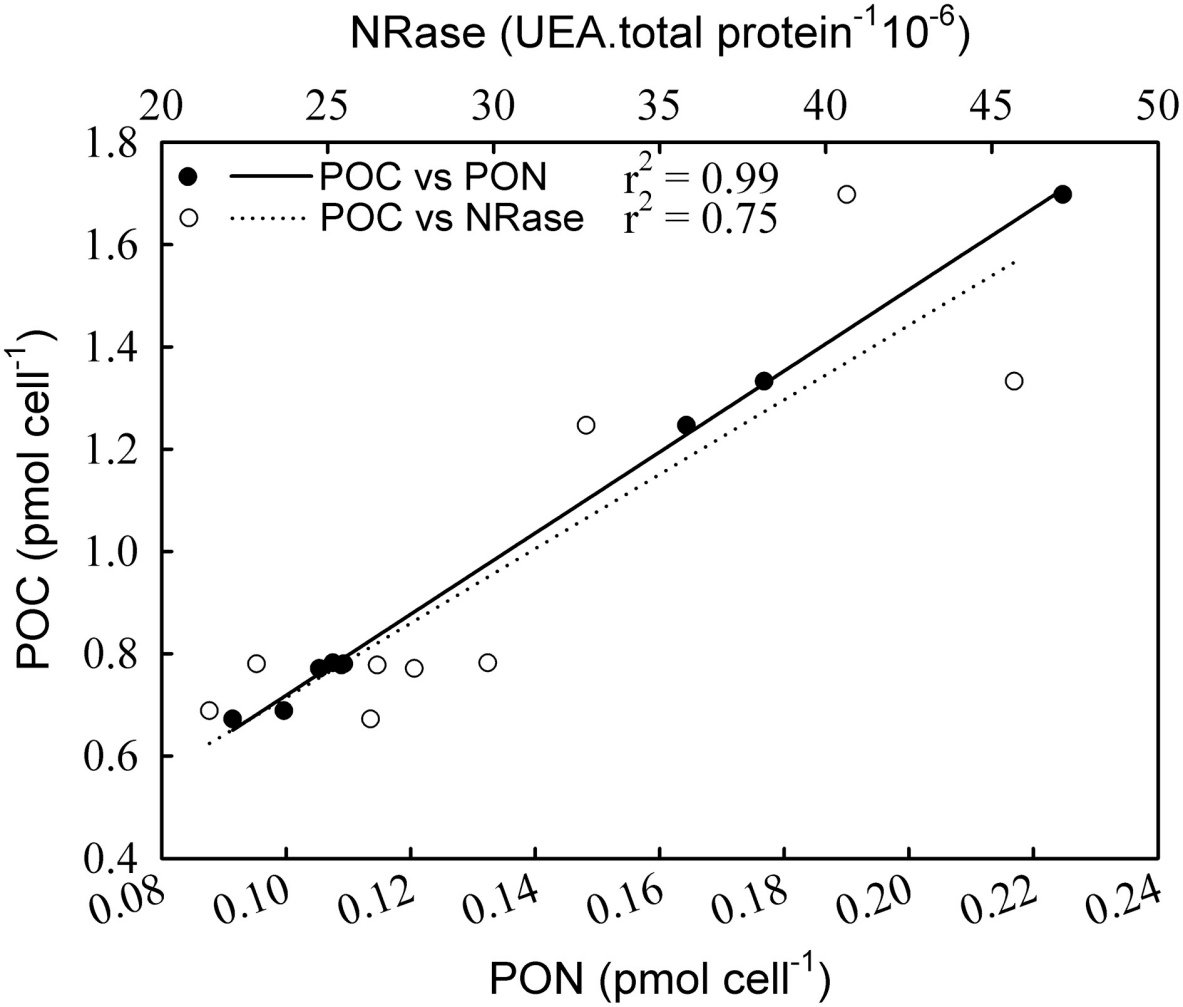
**Correlation of particulate organic carbon (POC) with particulate organic nitrogen (PON) and nitrate reductase (NRase) under nutrient replete conditions**.

**Figure 3:**
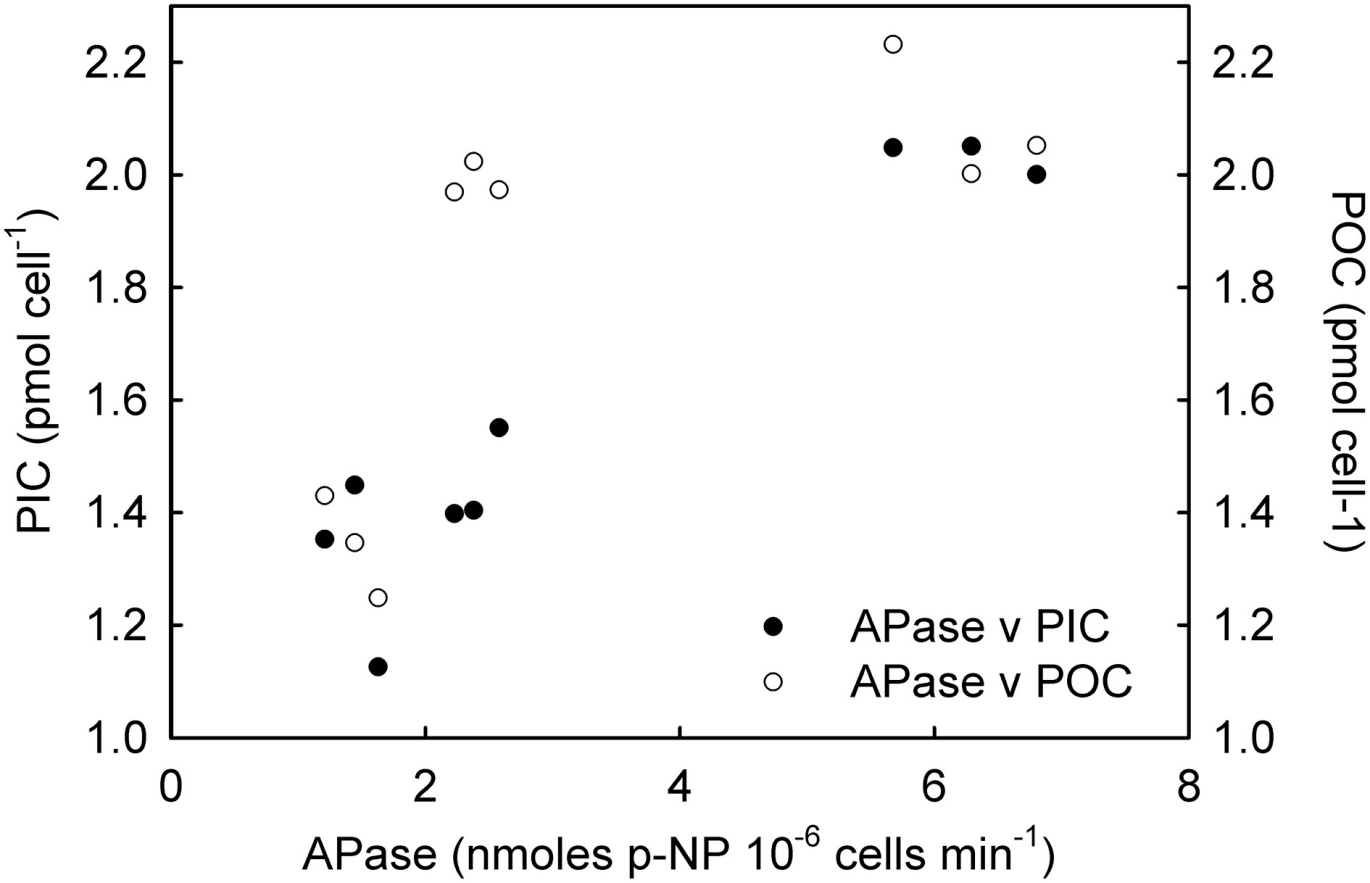
**Correlation of alkaline phosphatase (APase) with particulate organic carbon (POC) and particulate inorganic carbon (PIC) under phosphorus limited conditions**.

NRase activity was detected only under R conditions, and a linear increase in its activity [*r*^2^ = 0.79; *F*_(1,7)_ = 26.4; *p* = 0.01] was observed with increasing CO_2_ levels (Figure 1). This increase in NRase activity was accompanied by a simultaneous increase in the cellular POC quota [Figure 2; *r*^2^ = 0.74; *F*_(2,7)_ = 20.5; *p* = 0.02]. APase activity was only detected in the -P cultures and showed a maximum rate of 6.25 nmoles of *p*-NP 10^6^ cells min^−1^ at 561 μatm CO_2_. APase activity at 256 and 1314 μatm CO_2_ was 77 and 61% lower than at 561 μatm CO_2_ [Figure 1; *F*_(2,6)_ = 149.0, *p* < 0.001]. Cellular PIC quota showed a strong correlation with APase activity [Figure 3; *r* = 0.94; *F*_(1,7)_ = 59.1; *p* < 0.001] but not with cellular POC (Figure 3).

### Cell organic and inorganic matter quotas

**Figure 4:**
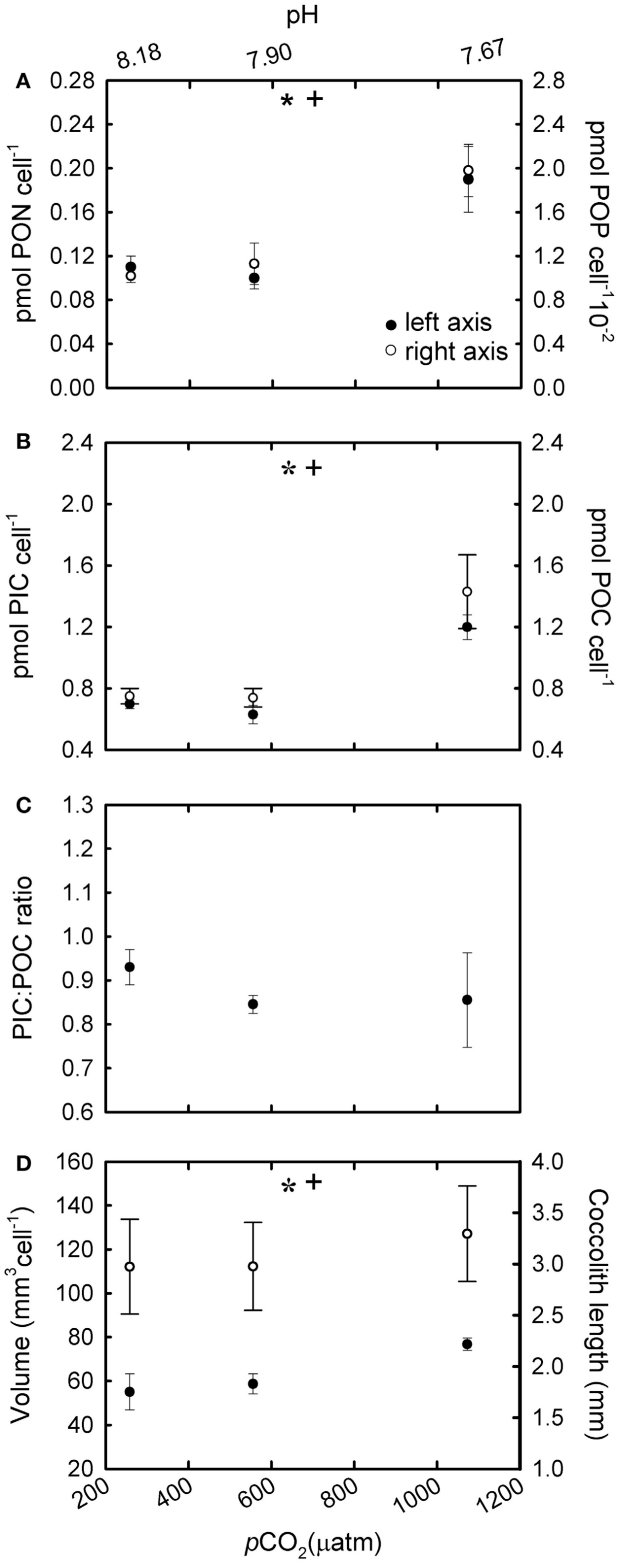
**The response of *Emiliania huxleyi* NZEH to different CO_2_ scenarios under nutrient replete conditions. (A)** Cellular PON quota, cellular POP quota; **(B)** Cellular PIC quota, cellular POC quota; **(C)** PIC:POC; **(D)** Coccosphere volume (μ m^3^) and coccolith length (μm). (^*^ and +) significant differences (*p* < 0.05) between CO_2_ levels within nutrient treatments from One-Way ANOVA analysis: left (^*^) and right(+) axis.

In R cultures, both cellular PON and POP quotas increased significantly with increasing CO_2_ [Figure 4A; *F*_(2,6)_ = 19.28, *p* = 0.002 (PON); *F*_(2,6)_ = 24.69, *p* = 0.002 (POP)]. Cellular PIC and POC quotas were ~70 and 90% higher respectively at the highest CO_2_ levels (555 and 1073 μatm) compared with cells grown under ~258 μatm of CO_2_ [Figure 4B; *F*_(2,6)_ = 80.37, *p* ≤ 0.001 (PIC); *F*_(2,6)_ = 10.65, *p* = 0.01 (POC)]. In addition, cellular POC quotas increased in parallel to PON quotas under different CO_2_ conditions [Figure 2; *r*^2^ = 0.99; *F*_(1,7)_ = 3596.4; *p* < 0.001]. The PIC:POC ratio did not change significantly with rising CO_2_ [Figure 4C; *F*_(2,6)_ = 1.41, *p* = 0.315] although a decreasing trend could be observed.

**Figure 5:**
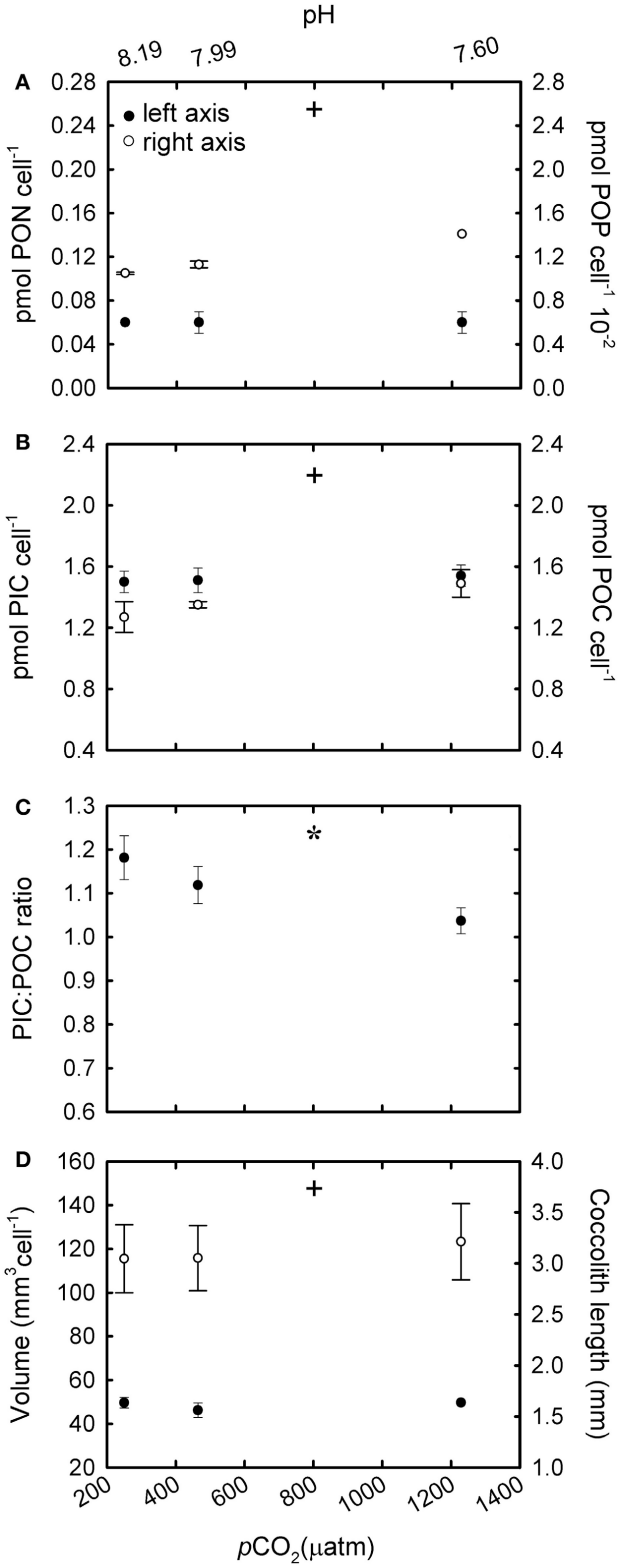
**The response of *Emiliania huxleyi* NZEH to different CO_2_ scenarios under nitrate limited conditions. (A)** Cellular PON quota, cellular POP quota; **(B)** Cellular PIC quota, cellular POC quota; **(C)** PIC:POC; **(D)** Coccosphere volume (μm^3^) and coccolith length (μm). (^*^ and +) significant differences (*p* < 0.05) between CO_2_ levels within nutrient treatments from One-Way ANOVA analysis: left (^*^) and right(+) axis.

In -N cultures, nitrate in the culture media was almost completely depleted after 8–9 generations (Table 1). The cellular PON quota was less than half the concentration of the R cultures [Table 2; *F*_(4,18)_ = 9.70, *p* ≤ 0.001]. Cellular PON quotas did not vary [Figure 5A; *F*_(2,6)_ = 1.22, *p* = 0.361] but POP quotas increased significantly [Figure 5A; *F*_(2,6)_ = 16.33, *p* = 0.004] with increasing CO_2_. Similarly, PIC quotas did no vary [Figure 5B; *F*_(2,6)_ = 0.35, *p* = 0.72], but POC quotas increased significantly with increasing CO_2_ levels [Figure 5B; *F*_(2,6)_ = 6.14, *p* = 0.035]. A significant decreasing pattern in PIC:POC was observed in -N cultures [Figure 5C; *F*_(2,6)_ = 9.07, *p* = 0.015] with increasing CO_2_.

**Table 2:** **Cell quota, coccosphere volume, coccolith length, and cellular ratios at the end of the experimental period (exponential phase for R cultures and exponential + nutrient limiting phase for -N and -P cultures)**.

**Nutrient and (pCO_2_) condition**	**PON (pmol cell^−1^)**	**POP (pmol cell^−1^ 10^−2^)**	**PIC (pmol cell^−1^)**	**POC (pmol cell^−1^)**	**PIC (pmol vol^−1^ 10^−2^)**	**POC (pmol vol^−1^ 10^−2^)**	**Volume (μm^−3^ cell^−1^)**	**Coccolith length (μm)**	**POC:PON**	**POC:POP**	**PON:POP**
	^*^	^*^	^*^	^*^	^*^	^*^	^*^	^*^	^*^		
R- 258.3	0.11 (0.01)	1.02 (0.06)	0.70 (0.03)	0.75 (0.05)	1.28 (0.15)	1.37 (0.12)	55.06 (8.17)	2.974 (0.463)	7.06 (0.13)	73.22 (4.23)	0.36 (0.5)
R- 555.6	0.10 (0.01)	1.13 (0.19)	0.63 (0.06)	0.74 (0.06)	1.07 (0.10)	1.27 (0.09)	58.68 (4.58)	2.977 (0.429)	7.32 (0.04)	66.45 (6.76)	9.08 (0.88)
R- 1073.1	0.19 (0.03)	1.98 (0.24)	1.20 (0.08)	1.43 (0.24)	1.57 (0.14)	1.85 (0.25)	76.82 (2.74)	3.292 (0.482)	7.56 (0.03)	72.05 (6.21)	9.53 (0.85)
		^*^		^*^				^*^		^*^	
-N- 250.0	0.06 (0.00)	1.05 (0.01)	1.50 (0.07)	1.27 (0.10)	3.02 (0.23)	2.56 (0.30)	49.64 (2.40)	3.042 (0.328)	23.2 (0.96)	121.34 (10.67)	5.24 (0.62)
-N- 463.5	0.06 (0.01)	1.13 (0.03)	1.51 (0.08)	1.35 (0.02)	3.27 (0.34)	2.92 (0.25)	46.22 (3.38)	3.055 (0.314)	23.8 (2.48)	119.65 (4.17)	5.08 (0.71)
-N- 1229.2	0.06 (0.01)	1.41 (0.00)	1.54 (0.07)	1.49 (0.09)	3.10 (0.12)	2.99 (0.15)	49.70 (0.33)	3.224 (0.364)	24.51 (0.48)	105.28 (1.19)	4.29 (0.11)
	^*^		^*^	^*^	^*^	^*^	^*^	^*^	^*^		
-P- 256.3	0.13 (0.02)	0.39 (0.03)	1.31 (0.17)	1.34 (0.09)	1.49 (0.11)	1.53 (0.03)	87.82 (7.04)	3.084 (0.328)	9.96 (0.53)	341.40 (14.72)	34.35 (2.29)
-P- 560.8	0.16 (0.01)	0.43 (0.22)	2.03 (0.03)	2.10 (0.12)	1.71 (0.04)	1.76 (0.07)	118.67 (2.03)	3.228 (0.312)	12.98 (0.23)	578.75 (277.01)	44.78 (21.81)
-P- 1313.7	0.18 (0.01)	0.59 (0.08)	1.45 (0.10)	1.99 (0.03)	1.39 (0.09)	1.90 (0.01)	104.46 (1.91)	3.427 (0.403)	11.00 (0.66)	342.5 (46.07)	31.34 (5.57)

Numbers in brackets refer to standard deviation (n = 3).

* Significant responses (p < 0.05) to pCO_2_ within each nutrient condition.

**Figure 6:**
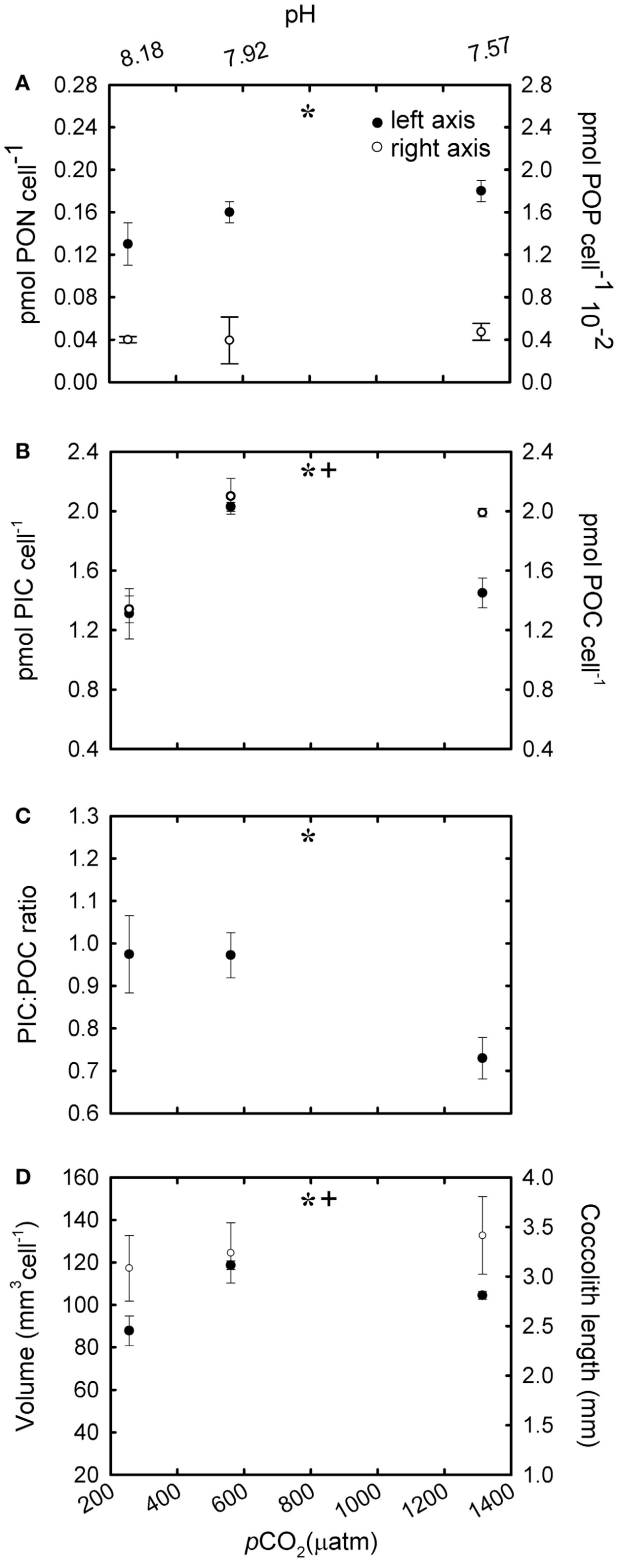
**The response of *Emiliania huxleyi* NZEH to different CO_2_ scenarios under phosphate limited conditions. (A)** Cellular PON quota, cellular POP quota; **(B)** Cellular PIC quota, cellular POC quota; **(C)** PIC:POC; **(D)** Coccosphere volume (μm^3^) and coccolith length (μm). (* and +) significant differences (*p* < 0.05) between CO_2_ levels within nutrient treatments from One-Way ANOVA analysis: left (*) and right(+) axis.

In -P cultures, phosphate in the culture media was almost completely depleted after 8–9 generations (Table 1). The cellular POP quota was less than half the concentration of the R cultures [Table 2; *F*_(4,18)_ = 7.39, *p* = 0.001]. Cellular PON quotas increased significantly [Figure 6A; *F*_(2,6)_ = 9.34, *p* = 0.014] but POP quotas did not vary [Figure 6A; *F*_(2,6)_ = 1.59, *p* = 0.279] with increasing CO_2_ conditions. The trend in cellular PIC quotas was not uniform and the most pronounced increase (>100%) was observed at 561 μatm of CO_2_ [Figure 6B; *F*_(2,6)_ = 37.03, *p* < 0.001]. Cellular POC quotas were ~50% higher at the two highest CO_2_ levels (~561 and 1314 μatm) compared with cells grown under 256 μatm CO_2_ [*F*_(2,6)_ = 10.742, *p* = 0.010]. In general, within each nutrient condition, the trend in PIC and POC quotas with CO_2_ varied when expressed as a function of coccosphere volume (Table 2). PIC:POC showed a decreasing pattern with increasing CO_2_ conditions [Figure 6C; *F*_(2,6)_ = 8.97, *p* = 0.016].

### Coccolith length and coccosphere volume

Under the highest CO_2_ level used, coccolith length was ~10% higher compared to the lowest CO_2_ condition for all the nutrient treatments [Figure 4D; *F*_(2,567)_ = 31.7, *p* < 0.001 (R); Figure 5D; *F*_(2,567)_ = 14.5, *p* < 0.001 (-N); Figure 6D; *F*_(2,567)_ = 43.8, *p* < 0.001 (-P)]. Coccosphere volume increased by ~39% over the CO_2_ range considered under R conditions [Figure 4D; *F*_(2,6)_ = 12.8, *p* = 0.007]. No differences in coccosphere volume were observed under -N conditions for all the CO_2_ levels tested [Figure 5D; *F*_(2,6)_ = 2.1, *p* = 0.207]. The highest coccosphere volume under -P conditions was observed at 561 μatm of CO_2_ [Figure 6D; *F*_(2,6)_ = 37.5, *p* < 0.001].

## Discussion

### Nutrient utilization: alkaline phosphatase and nitrate reductase

This is the first study to investigate *E. huxleyi* APase and NRase activities under varying CO_2_ levels. APase was only detected in -P cultures since APase activity is typically enhanced by phosphorus limitation. This enzyme allows phytoplankton to overcome phosphorus starvation by hydrolysing phosphate from esters in the dissolved organic phosphorus pool. APase activity showed a clear response to CO_2_ partial pressure, and its maximum activity was found at 561 μatm CO_2_ (Figure 1). Very little is known about the effect of CO_2_ in APase activities and phytoplankton (Tanaka et al., 2008; Endres et al., 2013), and the only study with *E. huxleyi* focused on the effect of pH (Xu et al., 2006). Since both CO_2_ and pH covaried in this study, the independent effect of these two parameters in APase activity cannot be discerned. As APase activity is known to be affected by changes in pH (Kuenzler and Perras, 1965), it is likely that pH is one, although maybe not the only, parameter affecting APase activity in this study.

A non-uniform response of APase activity to CO_2_ levels was observed, increasing from 256 μatm CO_2_ (*pH* = 8.18) to 561 μatm CO_2_ (*pH* = 7.92) but decreasing at the highest CO_2_ conditions (1314 μatm, *pH* = 7.57) (Figure 1). This decline in activity may be the result of a CO_2_ threshold exceeding the pH upper limit for optimum APase activity. In a study on the cyanobacterium species *Nodularia spumigena*, APase activity increased with CO_2_ (Endres et al., 2013). However, direct comparison across studies is not always possible because of the different CO_2_ levels applied. For example, the maximum CO_2_ conditions used in their study was ~700 μatm, probably still within the optimal pH range for this species. Additionally the thresholds of pH/CO_2_ tolerance cannot be generalized as APase optimum pH is species-specific (Kuenzler and Perras, 1965) and also dependent on the species biogeography (Yamada and Suzumura, 2010). The decline in APase activity at high CO_2_ (1314 μatm) suggests that the competitive ability of *E. huxleyi* to acquire phosphorus may be compromised in future more “acidic” oceans. However, the ecological implications of physiological results should be carefully considered given the high degree of genetic diversity among *E. huxleyi* strains (Iglesias-Rodriguez et al., 2006), the marked differences between strain maximum activities (Xu et al., 2010; Reid et al., 2011), as well as synergies with other levels of ecological organization.

NRase was only found in nutrient replete (R) cultures, when nitrate was present in the medium (Figure 1). Previous studies indicate a down-regulation of proteins involved in the acquisition and assimilation of inorganic nitrogen after nitrate depletion in *E. huxleyi* (Bruhn et al., 2010). Despite the presence of nitrate in the medium, NRase was absent when cultures grew in -P conditions. Since some regulatory mechanisms involved in nitrate assimilation include phosphorylation, phosphate limitation may be impairing this process (Beardall et al., 1998). NRase increased with rising CO_2_ levels under R conditions. The significant positive correlation between cellular POC and PON with NRase (Figure 2) could be explained by nitrogen and carbon metabolism being tightly coupled, such that a decrease in photosynthetic carbon fixation limits nitrogen assimilation (Hipkin et al., 1983; Turpin, 1991). It is possible that responses in NRase activity to ocean acidification may be species- and possibly strain-specific such as those found in higher plants (Fonseca et al., 1997; Matt et al., 2001) and phytoplankton species (Xia and Gao, 2005; Rigobello-Masini et al., 2006).

### Particulate inorganic and organic carbon quotas under nutrient-replete conditions

Both cellular POC and PIC quotas increased at high CO_2_ under nutrient replete (R) conditions (Figure 4B). This increase was accompanied by slight increases in coccosphere volume and coccolith size (Figure 4D). The observed increase in cellular POC quotas at high CO_2_ concentrations has been observed in previous studies (e.g., Riebesell et al., 2000; Iglesias-Rodriguez et al., 2008; Langer et al., 2009) and suggests that this species could be carbon limited in the present ocean. Considering the low affinity of RUBISCO for CO_2_ and the poorly efficient carbon concentrating mechanisms in *E. huxleyi* (Paasche, 2002), any increase in available CO_2_ would increase the speed of carbon fixation, and thereby cellular POC quotas (Barcelos e Ramos et al., 2010). The observed increase in cellular PIC quotas with rising CO_2_ levels is in accordance with other studies using *E. huxleyi* NZEH (Iglesias-Rodriguez et al., 2008; Shi et al., 2009) but in disagreement with Hoppe et al. (2011). Discrepancies between studies with the same strain have also been found with strains others than NZEH (Langer et al., 2009; Hoppe et al., 2011) and with parameters others than PIC such as growth rate. Several explanations could reconcile the different outcomes observed between studies using the same strain; for example, differences in the experimental set up (i.e., carbon manipulation methods, number of generations under the experimental conditions, differences in temperature or light). The method of pH manipulation does not seem to be driving the reported differences between experiments according to results by Hoppe et al. (2011) and Shi et al. (2009), which revealed similar PIC responses to increasing CO_2_ using bubbling with different CO_2_ partial pressures or acid/base addition. Regarding the number of generations exposed to the manipulation, Barcelos e Ramos et al. (2010) showed a rapid response of *E. huxleyi's* metabolic properties (including PIC quotas) in response to ocean acidification. Finally, any differences in laboratorial culturing conditions, e.g., temperature and irradiance could give different outcomes. For example, results from this study and those by Iglesias-Rodriguez et al. (2008) and Shi et al. (2009) cannot be compared with those by Hoppe et al. (2011) because the temperature used in the latter was 4–5°C higher. Additionally, synergistic effects can give different outcomes; for example, trends in cellular PIC can change when CO_2_ levels are combined with different temperatures (Borchard et al., 2011) or light levels (Zondervan et al., 2002).

Different outcomes between studies using the same strain could also be explained by genetic differences between the cultured strains themselves. For example, there is evidence of shifts in phenotypic and genomic properties of strains over time under continuous culturing in the laboratory (Lakeman et al., 2009). Also, the potential for a strain to evolve properties that deviate from those of its original phenotype it is known to increase with time of exposure under the new growth conditions (Lakeman et al., 2009). Thus, comparisons between studies using the “same” strain must be conducted with caution.

### Particulate inorganic and organic carbon quotas under nutrient-limited conditions

The ocean is a dynamic system and the physiological response of an independent species might differ depending on the combination of environmental parameters or stressors to which they are exposed, including nutrient limitation. Similarly to R cultures, the increase in POC quotas with increasing CO_2_ levels in both -N and -P cultures (Figures 5B, 6B) suggests that, under nutrient limitation, carbon may be rate-limiting for photosynthesis under the CO_2_ conditions commonly found in the open ocean. Our results are however, in disagreement with those using nitrogen (Sciandra et al., 2003) and phosphorous (Borchard et al., 2011) limiting conditions in a calcifying *E. huxleyi* strain, but in accordance with Leonardos and Geider (2005), using a non-calcifying *E. huxleyi* strain under high irradiance. These differences probably result from strain-specific responses and/or variations in laboratorial conditions. In contrast to cellular POC quotas, PIC quotas in -N and -P cultures showed a different trend to that observed under R conditions. Interestingly, in -N cultures, PIC quotas remained constant under the different CO_2_ conditions suggesting that nitrogen metabolism may be decoupled from calcification. However, under phosphate limitation a decrease in PIC quotas was observed under the highest CO_2_ conditions (1314 μatm). Interestingly, APase activity was tightly correlated with PIC quotas (Figure 3). However, elucidating the mechanistic effect of CO_2_ and phosphate limitation on calcification requires further work.

Different cellular elemental stoichiometry was found in the *E. huxleyi* NZEH strain under different environmental scenarios (Table 2). Stoichiometric mechanisms are known to play a very important role in defining the structure of the food web in aquatic ecosystems (Elser et al., 2000). Specifically, the biochemical composition of phytoplankton is known to affect grazing preference (Jones et al., 2002). Thus, changes in stoichiometry could influence the grazing-selection pressure, ultimately determining the prevalence of some strains *versus* others in future oceans. In addition, changes in cellular elemental stoichiometry should be taken into consideration when predicting the role of *E. huxleyi* in future biogeochemical cycles.

Unlike in the R manipulations, the PIC:POC ratios decreased with CO_2_ under both -N and -P conditions in the NZEH strain (Figures 5C, 6C), comparable to other *E. huxleyi* strains under nitrogen (Sciandra et al., 2003; Müller et al., 2012) and phosphorus (Borchard et al., 2011) limitation. This finding is important considering the geographic extent of oligotrophic oceanic waters and the importance of PIC:POC ratio in determining aggregate formation processes, properties and sinking velocities (Armstrong et al., 2002). The data presented here suggest that a N- and P-limited population in a high CO_2_ ocean would have a reduced PIC:POC ratio, which would increase the removal of CO_2_ as a combined effect of calcification (a source of CO_2_) and photosynthesis (a sink of CO_2_) (Frankignoulle et al., 1994). However, this could also reduce the net CO_2_ export to the deep ocean associated with the role of coccolith CaCO_3_ in forming aggregate as ballast (Armstrong et al., 2002; Biermann and Engel, 2010).

### Coccolith length and coccosphere volume

Although coccolith length was correlated with CO_2_, coccosphere volume was better correlated with nutrient availability (Figures 4D, 5D, 6D). Coccosphere volume increased with rising CO_2_ under R conditions, but an 11% decrease in coccosphere volume was observed under the highest CO_2_ level in -P cultures, similar to previous studies with the *E. huxleyi* strain PML-B92/11 (Borchard et al., 2011). It is interesting to note that, unlike R and -N cultures, changes in POC quotas were not associated with changes in coccosphere volume in -P cultures. Additionally, maximum PIC quotas were observed at 561 μatm CO_2_ while coccolith length showed the highest values at 1314 μatm CO_2_. Similarly, in -N cultures, increased coccolith sizes did not correlate with PIC quotas, which were constant at all CO_2_ conditions. These results suggest that under nutrient limitation and at the highest CO_2_ condition, *E. huxleyi* might hold coccoliths bearing less calcite than under the lowest CO_2_ levels.

### Implications

The *E. huxleyi* strain NZEH, whose coccolith production seemed to be resilient to ocean acidification, presents a different response to increasing CO_2_ depending on the nutrient condition. However, and similar to what was found under R conditions, this response still seems to be different to that observed in other *E. huxleyi* strains under -N and -P conditions. For the past few years, the ocean acidification community is urging the need for multiparametric experiments in order to gain a better insight into more realistic species-specific responses to environmental pressure. However, more strain-specific studies are also necessary in order to predict and understand the direction of future changes with a degree of certainty. This information is important to improve parameterizations in diagnostic and prognostic of global biogeochemical models.

### Conflict of interest statement

The authors declare that the research was conducted in the absence of any commercial or financial relationships that could be construed as a potential conflict of interest.
